# Hyperuricemia-induced complications: dysfunctional macrophages serve as a potential bridge

**DOI:** 10.3389/fimmu.2025.1512093

**Published:** 2025-01-28

**Authors:** Wenyi Gu, Jiajing Zhao, Yu Xu

**Affiliations:** ^1^ Department of Traditional Chinese Medicine, Shanghai Putuo Hospital of Traditional Chinese Medicine, Shanghai, China; ^2^ School of Pharmacy, Shanghai University of Traditional Chinese Medicine, Shanghai, China; ^3^ Engineering Research Center of Shanghai Colleges for Traditional Chinese Medicine New Drug Discovery, Shanghai University of Traditional Chinese Medicine, Shanghai, China

**Keywords:** hyperuricemia, inflammation, macrophage, treatment, oxidative stress

## Abstract

With the changes in modern life, hyperuricemia (HUA) has become a serious universal health issue, leading to rising morbidity and mortality. Characterized by elevated levels of UA, HUA has become an independent risk factor for gout, chronic kidney disease, insulin resistance, cardiovascular disease, nonalcoholic fatty liver disease, etc. As HUA is a metabolic syndrome, the immune response is likely to play an active role throughout the whole process. Moreover, macrophages, as an indispensable component of the immune system, may serve as a promising target for addressing hyperuricemia-induced inflammation. Along with their precursor cells, monocytes, macrophages play a key role in the pathogenesis of HUA, primarily through three specific aspects, all of which are associated with inflammatory cytokines. The first mechanism involves direct action on urate transporters, such as URAT1 and ABCG2. The second mechanism is the modulation of inflammation, including targeting toll-like receptors (TLRs) and the NOD-, LRR-, and pyrin domain-containing protein 3 (NLRP3) inflammasome. The third mechanism pertains to the effects on oxidative stress mediators. In this review, we summarize the underlying mechanisms of hyperuricemia, focusing on the effects of macrophages, therapeutic approaches, and clinical trials addressing hyperuricemia-caused dysfunction. Additionally, we highlight directions for future development, aiming to support future theoretical studies.

## Introduction

Hyperuricemia (HUA) is the second most prevalent metabolic disease worldwide, placing a significant burden on the public health system (1). According to the 2015–2016 National Health and Nutrition Examination Survey (NHANES), approximately 9.2 million US residents are affected by HUA (2), while its incidence in Europe is approximately 20% (3, 4). Recent global reports indicate that more than 40 million individuals suffer from HUA, with an incidence of about two per 1,000 person-years (2, 5). HUA results from an imbalance of uric acid (UA) synthesis and excretion, a typical metabolic disorder that affects multiple organs and influences the whole body’s systematic metabolism. In the human body, UA is categorized into two types based on saturation: soluble UA (sUA) and crystalline UA (in most cases, monosodium urate (MSU) crystals). High levels of either sUA or crystalline UA contribute to the development and severity of various metabolic syndromes, including gout, chronic kidney disease (CKD), insulin resistance (IR), cardiovascular disease (CVD), nonalcoholic fatty liver disease (NAFLD) (14, 15). According to existing clinical trial results, individuals with HUA are more susceptible to these conditions than healthy individuals (3).

Moreover, HUA is associated with increased rates of these metabolic disorders due to over-activation of innate immune functions, particularly the stimulation and infiltration of immune cells such as macrophages, monocytes, and neutrophils (4). Damage-associated molecular patterns (DAMPs), which are endogenous host-derived molecules produced or released by damaged and dying cells act as danger signals. They are responsible for alerting the host immune system to ongoing infections and promoting inflammatory responses in metabolic diseases (6, 7). DAMPs bind to specific receptors known as pattern recognition receptors (PRRs), which include toll-like receptors (TLRs) and the NOD-, leucine-rich repeat (LRR)-, and pyrin domain-containing protein 3 (NLRP3) inflammasome. PRRs have numerous downstream targets and are predominantly expressed in immune cells such as macrophages and neutrophils. The bond between DAMPs and PRRs activates the recruitment of those immune cells, initiating inflammation, releasing proinflammatory cytokines, nitric oxide (NO), and reactive oxygen species (ROS), and causing organ damage (8). UA is a member of the DAMP family and has been shown to activate macrophages, prompting their migration to lesions and triggering an inflammatory cascade, including the stimulation of NLRP3 inflammasomes, which subsequently exacerbates the inflammatory response. Furthermore, this can lead to inflammation in proximal tubule epithelial cells, endothelial cells, adipocytes, and other cells (9, 10). These cells, along with immune cells, play a crucial role in the occurrence and development of inflammation in metabolic diseases associated with HUA. Notably, macrophages are a heterogeneous population of immunocytes that occupy the majority of immune cells in autoimmune diseases. Their metabolic profiles include homeostatic maintenance, immune defense, and inflammatory responses (11). In metabolic diseases, macrophages act as front-line soldiers, protecting against pathogens. Extensive literature has identified the classic signaling pathways in macrophages during HUA and its multimorbid consequences, including the TLR4/NF-κB, JAK/STATs, and TGF-β/Smads signaling pathways, among others (12). Moreover, HUA may influence its comorbidities through oxidative stress in macrophages, as shown by many studies. This highlights the closely intertwined relationship between inflammation and oxidative stress.

To date, several studies have explored the correlation between HUA and macrophage. In this review, we comprehensively summarize previous findings on how UA contributes to these diseases, with a particular focus on macrophage modulation during disease progression. Additionally, we discuss interventions and clinical trials related to HUA and its associated comorbidities, concluding with our own recommendations.

## Overabundance of UA triggers macrophage and monocyte activation

### UA metabolism

**Figure 1:**
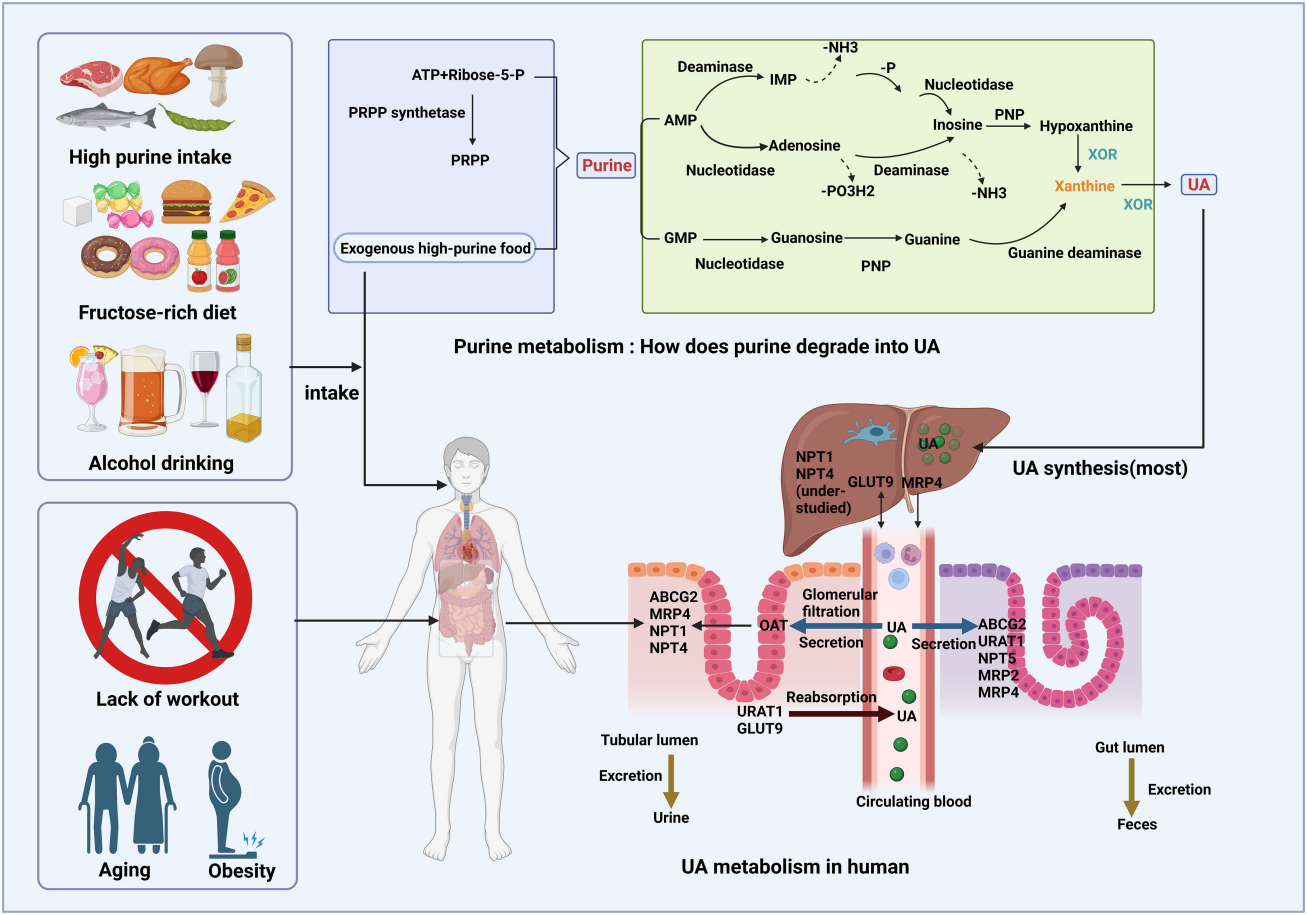
UA production, excretion, and reabsorption in humans. Due to the lack of uricase, UA serves as the final metabolite of purine metabolism in humans. Purine metabolism involves the nucleotide metabolism of AMP and GMP. AMP is converted to inosine via deamination or nucleoside cleavage, while GMP undergoes a similar reaction to form guanine. Inosine and guanine are subsequently converted into the common product xanthine, which is further metabolized to UA by XOR. UA synthesis primarily occurs in hepatocytes and, together with daily purine intake, undergoes a continuous cycle of excretion and reabsorption.

UA, also known as 7,9-dihydro-3H-purine-2,6,8-trione (C_5_H_4_N_4_O_3_), is the final product of human purine metabolism. In mammals, UA is regarded as an “excretion” product, while urea is used to eliminate excess nitrogen. Consequently, UA output reflects purine metabolism. As shown in **Figure 1**, purines have two sources: exogenous input and endogenous degradation. Exogenous purines primarily originate from daily food containing high purine levels, whereas endogenous purines are metabolized from adenine and guanine into UA, the end product of purine catabolism, through key enzymes such as xanthine oxidoreductase (XOR) and xanthine oxidase (XO) (13). Particularly, XOR is the rate-limiting enzyme in purine metabolism, responsible for producing UA and ROS in the host (14). UA metabolism operates as an internal circulation within the body, encompassing secretion, excretion, and reabsorption. However, the backbone of this cycle relies on membrane transporters such as organic anion transporter (OAT)1, OAT2, and OAT3; glucose transporter 9 (SLC2A9/GLUT9); urate transporter 1 (SLC22A12/URAT1); human sodium-dependent phosphate cotransporter type 1 gene (NPT1/SLC17A1); human sodium-phosphate transporter 4 (NPT4/SLC17A3); and ATP-binding cassette transporter subfamily G member 2 (ABCG2). These transporters (proteins) are collectively referred to as urate transporters. The liver is the main organ responsible for UA secretion, while UA deposition usually occurs in the kidney and intestine. After being filtered by the glomeruli, circulating UA is absorbed by OAT at the basolateral membrane of tubular epithelial cells. Subsequently, efflux transporters, such as ABCG2 at the apical membrane, secrete UA into the tubular lumen. In the intestine, the regulation of UA metabolism is critically influences by the deficiency of ABCG2 and the activity of GLUT9. Only a small portion of UA is finally excreted out from the body through the tubular and gut lumen as urine and feces. The majority is reabsorbed to control serum UA levels by URAT1 and GLUT9 (isoform2) at the apical membrane, returning UA to tubular epithelial cells. Meanwhile, GLUT9 (isoform1) at the basolateral membrane in the liver transports synthesized UA from hepatocytes into circulating blood (14, 15). As many of these transporters are also involved in glucose and lipid metabolism, disturbances in UA metabolism may lead to HUA and its associated comorbidities.

### Macrophage activities

Once danger stimuli are recognized by the body, circulating monocytes, the progenitor cells, migrate to the lesions and subsequently differentiate into macrophages. The properties of immune cells originating from the bone marrow are distinguished by their distinct surface markers (16). Ly6G+ is considered a marker for neutrophils, GR1+ and CD14+ are markers for myeloid-derived suppressor cells (MDSCs), and Ly6C+ and CD43+ are markers for monocytes. The unique macrophage marker is F4/80+, however, CD11b+ and CD68+ can sometimes be used as markers for non-F4/80+ macrophages.

In terms of origin, some macrophages are tissue-resident, such as Kupffer cells (KCs), while others may travel through the blood flow to settle in different locations, where they differentiate into microglial cells, osteoclasts, etc., after being exposed to specific physiological and pathological environments (17, 18). A significant characteristic of macrophages is their ability to transform into several phenotypes in response to local microenvironments. This change is referred to as the polarization of macrophages. Generally, based on their function, macrophages are categorized into a spectrum of proinflammatory, classically activated (M1 phenotype), and anti-inflammatory, alternatively activated (M2 phenotype) profiles. In most cases, during inflammation, macrophages initially manifest as M1 macrophages, releasing inflammogenic factors such as IL-1β, IL-6, IL-12, IL-18, IL-23, and TNF-α, along with the chemokine (IL-8), which aggravate the disease. As the disease progresses, M2 macrophages outnumber M1 macrophages, secreting cytokines such as IL-10 and TGF-β to inflammation. Regarding specialized surface markers, CD86+, CD11c +, and CD206+, CD163+ are classic indicators of M1 and M2 macrophage, respectively. Inducible nitric oxide synthase (iNOS) and arginase-1 (Arg-1) serve as signature molecules for M1 and M2 macrophage activation, respectively. Specifically, in the presence of nicotinamide adenine dinucleotide phosphate (NADPH) and oxygen, iNOS combines with l-arginine to generate NO, l-citrulline, and NADP^+^. Subsequently, NO plays a key role in producing proinflammatory mediators (19). On the other hand, Arg-1 competes with iNOS for free l-arginine in immune cells (20), which explains the two polarized states of macrophages. Among the typical transcription factors, NF-κB and (STAT)3 represent M1 and M2 macrophage polarization, respectively (21).

### UA-induced macrophage response

The signature of HUA is the elevation of serum UA levels without episodes of inflammation induced by urate crystals; many researchers have attributed asymptomatic HUA to the presence of sUA (22). Mounting evidence has verified that soaring sUA levels lead to the polarization of proinflammatory macrophages, such as through pathways involving the connection with TLRs and the priming and activation of NLRP3. TLRs, as PRRs, are massively expressed in renal cells and serve to defend against external pathogens. They recognize signature motifs, referred to as pathogen-associated molecular patterns (PAMPs), such as microbial products like lipopolysaccharide (LPS) (TLR4 ligand, a common proinflammatory agent) and DAMPs like UA, initiating an inflammatory cascade that influences the activation of immune cells. Typically, UA binds to TLR2/4 through URAT1, which then induces NF-κB to generate TNF-α and increases the expression of M1 macrophage markers, leading to the release of proinflammatory cytokines such as IL-1β, IL-6, and TNF-α in primary human macrophages (23).

Both the activation of TLRs and NF-κB, as well as the recognition of UA, strongly stimulate the activity of NLRP3 inflammasome, a pivotal player in metabolic flux. The NLRP3 inflammasome consists of NLRP3, the adapter protein apoptosis-related speck-like protein (ASC), and caspase-1. Particularly, NLRP3 recognizes danger signals and recruits downstream particles; caspase-1 instigates the maturation and secretion of IL-1β and IL-18, as well as the cleavage of gasdermin D, leading to pyroptosis, a form of cell death associated with a highly inflammatory state. ASC serves as a bridge connecting NLRP3 and caspase-1 (24). It has been reported that UA accumulation activates the NLRP3 inflammasome, eliciting IL-1β processing in macrophage-like J774.1 cells (25). On the other hand, Naip1, a member of the NLRB subfamily of NLRs, contains a baculoviral inhibitor of programmed cell death repeat domain at its N-terminal. It recognizes UA, initiates the assembly and activation of NLRP3 inflammatory complexes, and significantly increases IL-1β in expression in macrophages (26). However, recent study has unfortunately focused more on TLR-mediated NLRP3 inflammasome activation in MSU-induced kidney inflammation rather than in sUA-induced quiet renal diseases, highlighting the need for more data to elucidate and refine this mechanism.

Autophagy plays an important role in macrophages by regulating cytokine release, polarization, and phagocytosis. It is also linked to NLRP3 inflammasome activation in the context of HUA. For instance, a study using genetically modified *Uox*-KO rats demonstrated that HUA induced renal damage through the activation of autophagy via classical pathways such as AMPK and MAPK/ERK, which counteract mTOR, thereby priming NLRP3 inflammasome-triggered inflammation and macrophage infiltration (27). Furthermore, the underlying autophagic mechanism may be attributed to the p53 signaling pathway and the release of cathepsin B (28).

As another important participant in the progression of HUA, ROS accompanies the sUA surplus. Normally, ROS is assumed to be produced through XOR. However, a clinical study emphasized that UA directly generates ROS independently of XOR, although XOR is involved in the ROS scavenging process (29). Due to UA excess, ROS can exacerbate inflammation. Intriguingly, there has been debate over whether it is XOR-catalyzed UA that induces inflammation or XOR-derived ROS that triggers macrophage activity and inflammatory responses, such as IL-1β secretion upon NLRP3 inflammasome activation (30). Interestingly, some studies have also considered ROS a type of DAMP, for example, when mitochondria ROS leak into the cytosol and escape the cells (31). Indeed, existing data suggest that the accumulation of free oxygen radicals can produce IL-1β in cells, indicating that ROS is, correspondingly, an activator of the NLRP3 inflammasome.

**Figure 2:**
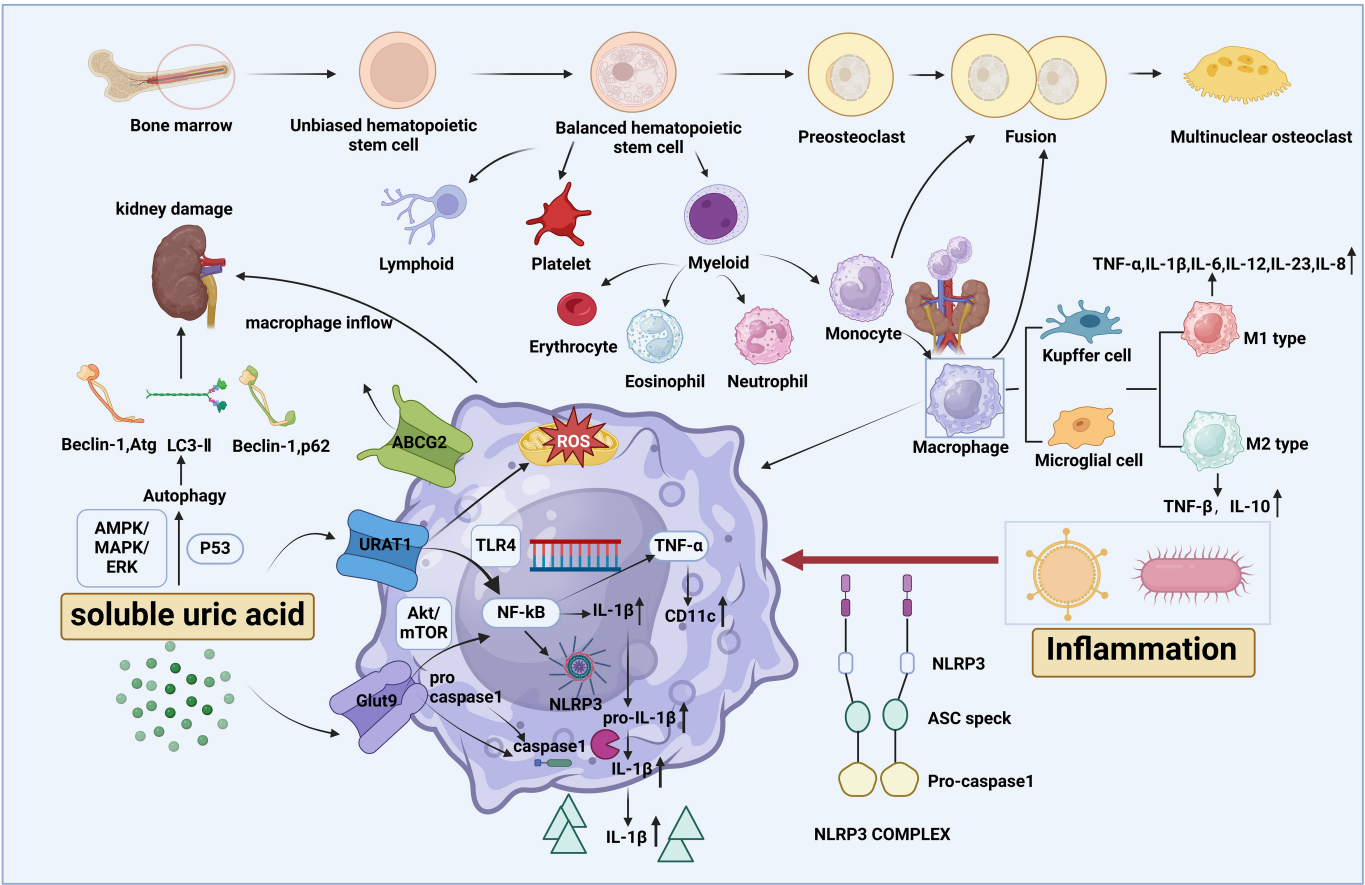
The origin of macrophages and the influence of soluble uric acid (sUA) on macrophage activity. Macrophages, along with their precursor cells, monocytes, are both myeloid cells derived from the bone marrow and share a common lineage with neutrophils. Through differentiation, macrophages are further categorized into specialized forms such as Kupffer cells and microglial cells. Based on cell function, macrophages are characterized into M1 and M2 phenotypes, each secreting specific cytokines. Soluble uric acid (sUA), a component of asymptomatic HUA, enters macrophages via urate transporters upon exposure to inflammation. Once inside, sUA activates NF-κB, promoting the release of inflammatory cytokines such as IL-1β, which is cleaved by caspase-1 from pro-IL-1β. This process polarizes macrophages toward the M1-like phenotype, primarily through the activation of the NLRP3 inflammasome and TLR4, while inducing mitochondrial ROS to amplify systemic inflammation. Additionally, sUA can trigger autophagy, leading to renal damage.

As the precursor cells of macrophages, monocytes stand at the frontline against external pathogens and tissue damage. Interestingly, however, there are two perspectives on the role of sUA in monocyte priming, depending on the extracellular environment. Under normal conditions, sUA directs monocytes to sites of inflammation and increases the quantity of circulating proinflammatory monocytes, thereby setting up a landscape for chronic inflammation, with GLUT9 being the target (32–34). Through histone ethylation, sUA phosphorylates proline-rich AKT substrate of 40 kDa (PRAS40), a junction between Akt and mTOR, further guiding NF-κB to commence the maturation of pro-IL-1β. This is then cleaved by activated caspase-1 into IL-1β, thereby triggering inflammation, which, along with the incremental formation of mitochondrial ROS, leads to monocyte pyroptosis. Another study verified the results of the above research, showing the effects of sUA on monocytes via the activity of Akt and its downstream PRAS40 (35). Nevertheless, under hypoxic conditions, sUA may reduce the inflammatory state of human monocytes by reducing ROS, IL-6, and IL-1β production (36). The above mentioned pathways are depicted in **Figure 2**.

In real life, we sometimes neglect the potential threat of sUA; however, when it progresses to its crystalline form, it becomes harder to address.

## Mechanisms underlying the exacerbation of macrophage-associated inflammation by MSU crystals

**Figure 3:**
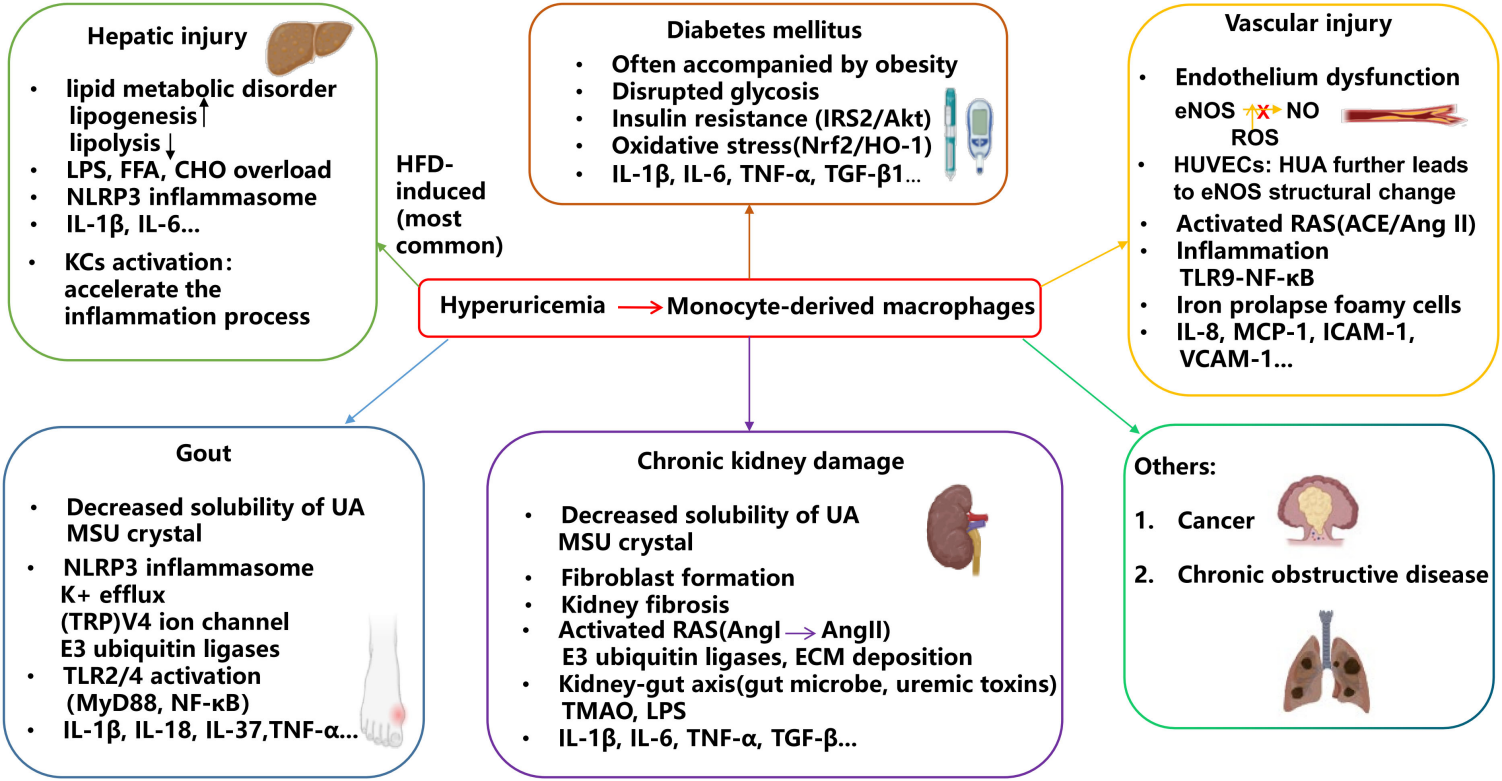
HUA contributes to the development of related diseases through macrophage-associated inflammation. Monocyte-derived macrophages play a key role in the inflammatory process associated with HUA comorbidities. As UA solubility decreases, MSU crystals form and accumulate in joints, triggering inflammation that leads to gout flares and chronic kidney damage. High UA levels induce oxidative stress, contributing to the progression of diabetes and vascular injury. Additionally, elevated UA can cause lipid dysfunction, potentially leading to hepatic injury.

About one-fourth of individuals with HUA will eventually develop gout, with MSU crystal accumulation in the joints and periarticular tissues (37). The transformation of sUA into MSU crystals depends on the decreased solubility of UA under certain environmental factors (temperature, humidity, long-term ultraviolet exposure) and pH levels (**Figure 3**). For example, a lower temperature may promote a greater possibility of MSU crystal deposition and formation at the same pH level (38). Cold intervention may not only accelerate the progression of MSU crystal precipitation but also induce inflammation through of NLRP3 priming and IL-1β secretion in macrophages (39).

### Macrophages and neutrophils in gout

It has long been agreed that a gout flare induced by MSU crystals is featured by pain and recurrence of acute inflammation with the penetration of neutrophils and macrophages (40). Particularly, the inflow of neutrophils into the joint is seen as the beginning of the acute gouty swelling characterized by the formation of neutrophil extracellular traps (NETs), whereas in the late stages of gout, they release anti-inflammatory cytokines to fight against disease progression (41). Additionally, macrophages are crucial in progressive inflammation (M1 type) and relief of gout (M2 type) (42). In the beginning, the making of chemokines such as C–C motif chemokine ligand 2–5 (CCL2–5) and C–X–C motif chemokine ligand 10 (CXCL-10) and cytokines like IL-1β, IL-6, and TNF-α is increased in macrophages, and progressively they also work with other immune cells to abolish the inflammatory responses (43).

Although the mechanism of sUA-associated inflammation in macrophages is incompletely understood, it is clear that the activation of NLRP3 inflammasome and TLR signaling are highly respected in both sUA and MSU crystal-induced macrophage activity, as shown in the **Supplementary Table S1**.

### NLRP3 inflammasome

The canonical NLRP3 inflammasome is an intracellular sensing protein complex whose activation practically comprises two steps—priming and activation. First, NLRP3 priming is started by the recognition of DAMPs, PAMPs, or cytokines by PRRs, and then NF-κB or other transcription factors or some transcriptional coactivators are upregulated, thus the transcriptional level of NLRP3 correspondingly rises. Sometimes ubiquitination, phosphorylation, and other posttranslational modifications of NLRP3 also rule the priming of NLRP3 for activation. In the second step, NLRP3 is activated by stimuli including DAMPs and ATP, largely attributed to changes in membrane potential, especially K+ efflux. Activated NLRP3 recruits downstream components to form the inflammasome complex. The structure of “sensor” NLRP3 is composed of three parts: an amino (N)-terminal pyrin domain PYD, a carboxy (C)-terminal LRR, and a central NBD-containing ATPase domain NACHT. PYDs are in charge of ASC recruitment; the LRR domain stabilizes the NLRP3 structure and senses danger signals; the NACHT domain hydrolyzes ATP to work with PYD to recruit ASC (24).

In the pathologic course of gout, a growing body of research evidence has revealed that MSU crystals as DAMPs first travel through lysosomes; afterward, the NLRP3 inflammasome forms a macromolecular conjugate to cleave procaspase-1 to caspase-1 and then promotes proteolytic initiation of IL-1β in macrophages; thus, gout is considered an NLRP3-dependent inflammatory disease. Many biological cofactors and specific modification processes happen during the activation of NLRP3. E3 ubiquitin ligases have been shown to interact with TLRs and facilitate NLRP3 inflammasome activation (**Figure 3**). Among them, Cullin1 induced the lysine 63-linked polyubiquitination of NLRP3 inflammasome; however, after sensing MSU crystals, the direct binding of SERTAD1 protein onto the NLRP3 inflammasome inhibited Cullin1 from interacting with the NLRP3 inflammasome and reduced the expression of IL-1β and IL-18 in macrophages. In terms of mechanism, when the lysine 63 deubiquitination of NLRP3 is suppressed, the maturing process of caspase-1 and IL-1β is halted, thus interdicting MSU-induced inflammation (44). Clonal hematopoiesis of indeterminate potential (CHIP) is highly associated with gout prevalence, and TET methylcytosine dioxygenase 2 (TET2) is among the most frequently mutated genes in the CHIP cases based on the database from MGBB and UKB. Urate crystals were found to enable TET2-mutant CHIP to worsen the inflammatory profiles in macrophages through the stimulation of NLRP3 and subsequent secretion of IL-1β (45). Moreover, TET2 mutations may dysregulate the release of mitochondrial DNA through cGAS signaling in macrophages during CHIP (46).

K^+^ efflux is a common upstream signaling event mainly triggered by NLRP3 stimuli-ATP and other DAMPs (**Figure 3**). The drop in cytosolic K+ concentration may induce a change in NLRP3 localization and structure. MSU crystal opens the Kv1.5 channel, one K+ channel encoded by *KCNA5*, by enhancement of Hsp70 protein to facilitate K+ efflux. Following that, ASC oligomerization and speck formation activate the NLRP3 inflammasome, and the expressions of caspase-1 and IL-1β are upregulated in macrophages (47). NIMA-related kinase (NEK)7 is ownstream of K^+^ efflux and also serves as an NLRP3 activator. In this process, NEK7 forms a bond with the LRR domain of NLRP3 and induces NLRP3 to expose its hidden PYD domain. After that, with the inclusion of ASC and caspase-1, the NLRP3 oligomer complex is formed. However, the cut down on the connection between NEK7 and the NLRP3 inflammasome will ultimately alleviate the inflammatory state of MSU crystal-presented macrophages (48). Independent of K+ efflux signaling, the transient receptor potential V4 ion channel on macrophages is opened to identify and phagocytose MSU crystals, and then the NLRP3 inflammasome is initiated, followed by IL-1β and ROS release *in vivo* and *in vitro* (49) (**Figure 3**).

A high concentration of glucose may drive glycolysis, which has an important impact on the NLRP3 inflammasome oligomerization. In response to MSU crystals, glucose transporter 1 (GLUT1) could upregulate glycolysis and glucose uptake and then instigate the formation of the NLRP3 inflammasome complex and IL-1β secretion in macrophages; there was a rise of GLUT1 on the neutrophil surface membrane (50). In addition to glucose uptake, the hindrance of the tricarboxylic acid (TCA) cycle or the direct inhibition of immunoresponsive gene 1 may lead to a robust decrease in free radicals, M1-polarized macrophages, and IL-1β and TNF-α levels against gout (51).

IL-37 is widely existent in primates but is poorly understood in its mechanism of regulating diseases. It may bind to the IL-18 receptor subunit IL-18Rα to inhibit the maturation of the NLRP3 inflammasome and pyroptosis, restoring gouty arthritis (52). Moreover, it can inhibit the release of inflammatory factors such as iNOS, polarize macrophages to the M2 phenotype, and let macrophages behave as “silent” noninflammatory phagocytosis to MSU crystals, which makes it a particularly suitable target for the treatment of chronic gout (53). Moreover, its restoration of cellular membrane permeability and cellular metabolism is also considered an influencer of inflammation by regulation of LPS-induced TLR4 signaling (54).

### TLR stimulation

One recent case-control study indicated that high expression of TLR4 is positively correlated with the prevalence of gout (55). TLRs family, particularly TLR2 and TLR4, are believed to stimulate the myeloid differentiation factor 88 (MyD88) and modulate macrophage phagocytosis of urate crystals. Also, TLRs activate NF-κB and the NLRP3 inflammasome, leading to the release of IL-1β and secondary proinflammatory cytokines and chemokines, such as TNF-α, in macrophages to exacerbate MSU-induced inflammation (56) (**Figure 3**). In the presence of MSU crystals, triggering receptors expressed on myeloid cell-1 (TREM1) (57), myeloid-related protein-8/14(MRP-8/14) (58), histone deacetylase (HDAC), and various factors interplay with TLR signaling in macrophages. In particular, HDAC enzymes incite posttranslational modifications of their target substrates to activate gene transcription via enhanced deacetylation activity and intracellular trafficking ability. In immune diseases, HDAC deacetylates NF-κB, activates NLRP3 inflammasome, and induces inflammatory factor secretion. HDAC3 (class I HDAC) deletion may restrict the recruitment of neutrophils to the lesions and the activity of NF-κB, polarize macrophages to an anti-inflammatory state, and mitigate the level of IL-1β, TNF-α, and IL-6 mediated by the TLR2/4 pathway to prevent MSU crystal-associated joint swelling (59). Bone-resident macrophages have the potential to differentiate into osteoclasts. The concentration of HDAC6 (class II HDAC) in macrophages is upregulated in stimulation with MSU crystals, followed by the priming and activation of the NLRP3 inflammasome, promoting osteoclastogenesis in gout (60). Cyclic GMP-dependent protein kinase II expression is upregulated in response to MSU crystals in macrophages and triggers the polarization of the proinflammatory phenotype of macrophages and uric salts phagocytosis dependent on TLR2 (61). Cartilage injury accelerates the precipitation of MSU crystals and the process of external and internal pathogenic invasion in gout. Type II collagen, one kind of extracellular matrix (ECM) cartilage fragments, formed a complex with urate crystals and stimulated MSU crystal phagocytosis of macrophages, expedited the level of IL-1β, TNF-α through the ITGB1-dependent overactivated TLR2/4 pathway, and provoked oxidative stress, resulting in the severity of gout (62). Well-known for preserving cartilage integrity, recombinant human proteoglycan-4 exerts its anti-gout properties by restraining phagocytosis of MSU crystals, NF-κB nuclear translocation, and NLRP3 inflammasome activation in macrophages due to its interaction with CD44 (mainly) and TLR2/4 (partially) (63).

Moreover, most innate immune cells during gout attacks circulate around the peripheral nerve fibers and are regulated by the neural system. Dorsal root ganglion (DRG) neuron activity, sometimes interacting with CGRP+ nociceptors, influences the polarization of macrophages and the release of proinflammatory cytokines in the presence of MSU crystal-related sFRP2, which polarizes M1-like macrophages and enhances inflammatory responses (64, 65). However, the neural system is very delicate, thus the relationship between neurons and MSU crystal-induced inflammation remains an understudied topic.

## HUA causes comorbidities with macrophage dysfunction

### Chronic kidney damage

Escalated UA level is a parameter of the injured kidney. In clinics, UA excretion malfunction and extra-renal UA excretion are observed in many CKD patients (66, 67). The development of renal damage induced by HUA is normally believed to be promoted by crystalline UA only, as evidenced by the detection of urate crystal granuloma and crystalluria (68). The severity of kidney damage can be conferred by macrophage infiltration and accumulation into the renal parenchyma. Those macrophages are responsible for either tissue remodeling or organ damage.

Kidney fibrosis is a representative renal disease characterized by fibroblast heterogeneity, and those ECM-enriched fibroblasts induce macrophage polarization and proliferation in diseased kidneys, such as an expansion of Spp1+ and F4/80+ macrophage signatures (69, 70). Vitronectin, as one ECM protein, is enriched in macrophages to form the fibrogenic niche, thereby leading to fibroblast activation and lesion, along with the polarization, proliferation, and migration of M1-like macrophages to direct kidney fibrosis (71). Integrins influence cell adhesion in injured kidneys through both cell–matrix and cell–cell interaction, binding with focal adhesion kinase to drive the polarization of M2 macrophages, which are believed to act as profibrotic agents. UA directly promotes the expression of integrin αM in macrophages, leading to an increase in the M2 macrophage population and a decline in renal function (72). Additionally, macrophage-induced inflammatory responses in HUA-related renal damages cooperated with the renin-angiotensin system (RAS) and crosslinked the gut–kidney axis in a large part (**Figure 3**).

#### RAS

In the Chinese community population, individuals with hypertension are more subject to CKD with a threefold increase in the risk, correlating with more M1 macrophage infiltration compared to those with normal blood pressure (73). RAS is an unbiased hormonal cascade that regulates cardiovascular and renal functions. Aspartyl protease renin cleaves angiotensin I (AngI) from its inactive substrate angiotensinogen (AGT) to modulate the RAS circulation. AGT is further cleaved by two enzymes: renin and angiotensin-converting enzyme (ACE). Renin is produced in the renal afferent arterioles and initiates the RAS cascade, which culminates in the production of AngI. ACE then is responsible for converting AngI into angiotensin II (AngII), which exerts negative feedback on the release of renin into the circulation (74). AngI and AngII participate in the inflammatory process during renal fibrosis, and mast immune cells produce chymase to act on AngI to generate AngII, the real main effector of the RAS with its non-hemodynamic properties (75).

In diseased kidney models, AngI receptors on macrophages can suppress the heightened expression of proinflammatory cytokines, whereas AngII infusion is intimately associated with the inflammatory responses, particularly the interaction between TLR4 and the NLRP3 inflammasome. Additionally, AngII triggers polyubiquitination of certain kinases, such as mitogen-activated protein kinase 7, exacerbating the local fibro-inflammatory environment (76). Thus, AngII receptor blockers such as losartan and fimasartan are classical drugs to treat CKD with hypertension due to urine albumin-to-creatinine ratio (UACR) reduction. Uric salts induce the activation of the RAS, and later on, host immunity responds to elevated blood pressure with the start-up of inflammation and recruitment of immunologic cells. For example, AngII inhibition abrogated MSU-induced inflammation targeting NLRP3 priming and activation and associated cofactors’ expressions *in vivo* and *in vitro* (77). TGF-β1, a key player in progressive kidney diseases that is partially prompted by AngII, incites cell proliferation, apoptosis, and immune responses. MSU crystals enhance the transcriptional level of Alpha-kinase 1 (ALT1), an indicator of inflammation in various immune diseases, which further augment increases in the expression of renin, TGF-β1, and IL-1β, exacerbating kidney injury (78). Blockade of angiopoietin-2 (Angpt2), a Tie-2 receptor, may alleviate ongoing inflammation and change the microenvironment during kidney fibrosis by downregulating TNF-α and TGF-β1, and facilitating the programmed death of hyaloid vascular endothelial cells with the help of Wnt, a sensor that is activated in response to kidney injury, from macrophages (79). Diabetes is often viewed as a coexisting disease of hypertension. Both shared similar risk factors, particularly inflammation and oxidative stress, which are largely attributed to RAS-induced endothelial dysfunction. AngII receptor inhibition is likely to act on the activation of mineralocorticoid receptors to turn UACR to normal standard (30 mg/g), represented by finerenone (80). Since the three interact with each other, the medical term “hypertensive diabetic CKD” is used to describe the combination of the three. Activated RAS contributed to glomerular hyperfiltration, a sign of diabetes, and a blast of inflammatory-associated cytokines via intracellular signaling pathways such as protein kinase C (PKC), JAK/STATs, and TGF-β/Smads. Dipeptidyl peptidase-4 (DPP-4) is a serine protease predominantly present in renal cells and plays a significant role in the progression of diabetic CKD, alongside sodium-glucose cotransporter (SGLT). DPP-4 inhibitors have been shown to exert reno-protective effects in diabetic kidney disease by regulating inflammatory reactions, which are associated with the polarization of M1/M2 macrophages and the transcription of NF-κB. AngII-mediated kidney DPP-4 assembles CD11b^+^F4/80^lo^Ly6C^−^ resident macrophage infiltration in rodent models, while its inhibition preserves the expression of Arg-1 protein and IL-2 and IL-9 (81).

#### Kidney-gut axis

The kidney is responsible for the whole process of UA metabolism, while the intestinal tract also plays a partial role in UA excretion. Therefore, there is cross-talk between the kidney and gut in terms of HUA. CKD may lead to the retention of nitrogen waste, promoting gut dysbiosis and compromising intestinal permeability, which results in the excessive production of opportunistic pathogens and their metabolites, such as uremic toxins like trimethylamine *N*-oxide (TMAO) and indoxyl sulfate (IS), further contributing to renal injury progression due to an increased inflammatory response (82). Specifically, TMAO promotes the accumulation of intestinal M1 macrophages through NLRP3 inflammasome activation, resulting in kidney fibrosis (83). IS, in combination with microbial molecules LPS, exacerbates the inflammatory status in macrophages, accompanied by elevated levels of TNF-α and IL-1β, thereby accelerating the progression of CKD (84). Interestingly, UA is considered by some as a uremic toxin, as uremic toxins are defined as molecules that damage renal function over time. Similarly, UA accumulation negatively impacts the kidney. On the other hand, a growing body of literature indicated that various gut microbes can mediate UA metabolism because they view UA and purine as carbon and energy sources anaerobically to combat inflammation, especially with the involvement of *Lacticaseibacillus*, *Akkermansia*, *Ruminococcus*, and *Clostridium*, as well as a rise in the production of bioactive molecules such as short-chain fatty acids (SCFAs). Impaired intestinal structure and gut microbiota alternations, particularly the increase in the Firmicutes phylum and decrease at the phylum of Bacteroidetes, may lead to the exacerbation of HUA and CKD associated with activation of the NLRP3 inflammasome and TLR4/MyD88/NF-κB signaling, inflammatory cell infiltration, and macrophage polarization. The mechanism may lie in the intestinal amino acid metabolism disturbance, renal UA metabolism perturbation, and oxidative stress induction (85–87). Moreover, probiotics have shown great potential in remodeling the gut microbiota, enriching SCFAs, and restraining inflammatory signals. In particular, certain strains of *Lactobacillus*, including *Lactobacillus fermentum* F40-4, *Lactobacillus reuteri* TSR332, and *Lactobacillus fermentum* TSF331, could degrade purine by regulation of urate transporters, drive the intestinal microbiome alternations, and initiate host immune responses against inflammation (88, 89). Lastly, many other dietary interventions, like inulin and vitamin C, restored imbalanced gut microbiomes and alleviated inflammation to deal with renal injury. Chlorogenic acid, as a representative ingredient found in many vegetables and fruits, could modulate the purine metabolism and glutamate metabolism, cease the secretion of proinflammatory factors such as LPS and NLRP3 via TLR4/MyD88/NF-κB signaling, and maintain the function and integrity of gut barrier through increasing the abundance of SCFA-producing bacterial genera such as *Bacteroides* and *Alistipes* (90).

### Hepatic injury

HUA has been linked to the severity of liver damage, as evidenced by elevated ALT and AST levels. The liver is the primary organ responsible for UA production, establishing a close relationship between HUA and hepatic injury, possibly due to the by-products generated during UA metabolism, particularly ROS (91). Addiitonally, UA overload can promote both inflammation and lipoprotein oxidation in liver tissue (92). As UA concentration rises, macrophages may infiltrate the liver and polarize to the M1 phenotype, thereby exacerbating liver damage and impairment (**Figure 3**).

#### KC activation

KCs are the residential hepatic macrophages that originate from yolk sac-derived precursors during embryogenesis. They protect against exogenous substances such as cell debris, pathogens, or apoptotic cells as the immunological barrier in the liver. As one kind of macrophage, they share certain similar profiles with nonresidential macrophages, such as the action of polarization in different microenvironments. In the context of HUA, they respond immediately to the pathogenic stimulus and are responsible for driving the transformation of Ly6C^+^ monocytes into M1-like macrophages, serving as initiators and main forces of the immune response at the initial stage of liver injury (93). The activation of the TLRs, NLRP3 inflammasome, and NF-κB, as well as the secretion of cytokines like IL-1β, TNF-α, and IL-6, are often probed in KCs during disease progression (94). A high-fat diet (HFD), rich in low-density lipoprotein (LDL), is an efficient way to create a foreseeable hepatic inflammation associated with lipid overload and UA metabolism disorder. Additionally, an increase in inflammation-provoking factors in KCs has been observed. Apigenin, a regulator of lipid metabolism, can recruit KCs to the lesions and mitigate the activity of the NLRP3 inflammasome, as well as the release of IL-1β and IL-18, by inhibiting XO activity in HFD-induced mice (95). In HFD-induced liver damage, the activation of macrophage scavenger receptor 1 on KCs not only mediates lipid uptake and accumulation via the JNK signaling pathway but also leads to the release of TNF-α, IL-6 (96).

#### Monocyte-derived macrophage-induced hepatic inflammation and lipid disorder

In addition to the KC activation, during hepatic injury, monocyte-derived macrophages are recruited, and inflammatory factors are secreted, further worsening the situation. These macrophages are responsive to a variety of stimuli, including bacterial endotoxins such as LPS and hepatotoxic lipids like free fatty acids (FFAs) or cholesterol. Rocketing serum LPS level is a hallmark of hepatic inflammation, and macrophage polarization toward the M1 type, coupled with impaired autophagy, can accelerate the inflammatory response in macrophages through its interaction with TLR4 and the activation of NLRP3 inflammasome (97). LPS is also a biomarker of gut dysbiosis; therefore, the overall intestinal barrier function is closely related to the hepatic inflammatory profile. Antimicrobial peptides, primarily produced in intestinal epithelial cells and macrophages, protect the innate immune system from microbial infections and intervene with the growth of detrimental bacteria in the gut lumen. Cathelicidin-related antimicrobial peptides target the degradation of LPS to inhibit the activation of TLR4 and the NLRP3 inflammasome. Its delightful outcome in relieving liver damage included the attenuation of lipid overload and oxidative stress, the downmodulation of UA-triggered IL-1β and XO levels, and improvement in microbial structure, particularly *Akkermansia* and *Bacteroides* (98). Likewise, *Limosilactobacillus reuteri* HCS02-001 restrained the entry of LPS into circulating blood and rescued HUA-induced liver inflammation through the TLR4/MyD88/NF-κB pathway (99). Excess FFAs and cholesterol can cause the formation of hepatic foamy macrophages, encourage KCs to aggregate lipogranulomas, andpromote their inflow to the lesions, thereby exacerbating the inflammatory status. Ganz et al. reported that high cholesterol and fructose increase the risk of liver injury through excessive lipid deposition, overproduction of danger signals such as UA, activation of inflammasomes, and the transformation of macrophage phenotypes (100). High levels of fructose are a causal factor for elevated UA concentrations and lipid metabolism disturbances in various liver diseases. In the liver, fructose first acts on AMP, then affects XO to produce excessive UA, eventually amplifying inflammation and the redox state in the host. Conversely, UA may yield more endogenous fructose by regulating fructokinase and aldose reductase (a key enzyme in glucose metabolism) (101). However, UA-induced fructose metabolism could be independent of fructokinase. Cytosolic endotoxemia prompted by fructose may activate TLR/MyD88 signaling in hepatic macrophages to mitigate the inflammatory state (especially TNF) and lipid deposition (102). Addiitonally, fructose often influences the fecal microbiota community, total SCFAs, and intestinal barrier function (103).

### Vascular injury

Since CVD is among the leading causes of mortality, especially the attack of heart disease, the relationship between HUA and CVD has been well-studied during the last few years. In the URic acid Right for heArt Health project, researchers found that UA has a positive correlation with various kinds of CVD, including fatal myocardial infarction, heart failure, and stroke (104). Nitric oxide (eNOS) accessibility and oxidative stress are key players in vascular dysfunction accompanied by HUA (**Figure 3**).

First, NO, produced by eNOS, is the essential molecule to maintain vascular homeostasis. With the addition of the deficiency of UA degradation in the vascular endothelial cells, the regular NO formation reaction is disrupted due to the changing of the structure of eNOS, aggravating the illness (105). Moreover, HUA encourages the interaction between endothelial cells and immune cells through chemokines, adhesion molecules, and other cofactors, including IL-8 and monocyte chemotactic protein-1 (MCP-1), in regard to cholesterol efflux. The increase in IL-8 may bind with CXCR2, prompting the generation of NETs in neutrophils and may further license macrophages for producing proinflammatory cytokines through the TLR9-NF-κB pathway (106). MCP-1 may bind with CCR2 in response to low HDL-C levels in the vascular plaque to invite macrophage and monocyte infiltration (107). Also, UA induces oxidative stress that binds to NO and encourages endothelial dysfunction and apoptosis through PKC-mediated eNOS activity and NO production, participating in the formation of atheromatous plaque (108). This oxidative stress will drive the oxidation of LDL in the subendothelial layer of the vascular endothelial cells into oxidized LDL (oxLDL), which may give rise to inflammation and the transformation of monocytes and macrophages into foam cells. Foam cells further amplify the inflammatory process by secreting proinflammatory cytokines and chemokines, which recruit leukocytes to produce TGF-β, eventually leading to more severe, end-stage cardiovascular injury (i.e., atherosclerosis) (109). Numerous studies have investigated the role of iron deposition in converting macrophages to foam cells during atherosclerosis. Recently, researchers discovered that HUA exacerbates oxLDL-induced iron prolapse and redox state, driving ferroptosis in macrophages and converting them into foam cells via the Nrf2/SLC7111/GPX4 signaling pathway, thereby promoting CVD (110). However, studies have shown that UA can also act as an antioxidant to inhibit ROS production and the formation of monocyte-generated foam cells caused by oxLDL, ultimately slowing the progression of malignant pathogenic invasion (111).

Fructose not only correlates with kidney damage but is also a key player in hypertension, particularly when combined with salt. These two factors contribute significantly to the global increase in CVD risk, accompanied by changes in UA levels and intestinal microbiota. Limiting high fructose and salt intake may improve UA circulation, reduce inflammatory, and decrease the relative abundance of *Lactobacillus* and *Bifidobacterium* via amino acid pathways, ultimately leading to blood pressure-lowering effects (112). A previous study consistently indicated that reducing the intake of a Western diet, which is rich in fructose, prevents the overstimulated XO ability, leading to decreased production of UA and reactive oxygen species, as well as a reduction in immune cell adhesion and the polarization of M1 macrophages (113).

Moreover, elevated serum UA is associated with left ventricular hypertrophy and diastolic dysfunction, which are significant vascular concerns. Allopurinol alleviated left ventricular hypertrophy caused by a Western diet, showing a significant effect in suppressing the TGF-β1/Smad signaling cascade, ultimately reducing the polarization of M1 macrophages and relieving inflammation (114).

### Diabetes

Glucose metabolism disorders, which are normally known as diabetes, are characterized by IR. Various epidemiological researchers have claimed that decreased serum UA levels are a detached benefit for the treatment of diabetes (115). The induction of diabetes from HUA is possibly due to the overproduction of purine by way of increased activity of the hexose monophosphate pathway shunt. Glycolysis is a critical step in glucose metabolism, and the disruption of glycolysis results in the overproduction of glucose-6-phosphate through the above pathway shunt, which explains why diabetic patients usually have high levels of UA (116) (**Figure 3**). Moreover, the lack of glycerol in glucose metabolism may result in the production of proinflammatory cytokines and M1 macrophages, as well as a rise in UA levels based on feedback (117). The relationship between plasma glucose and UA levels is typically presented by a classic “bell-shaped” curve, where UA levels initially rise and then decrease, coinciding with an increase in glucose concentration, as indicated by elevated glycated hemoglobin A1c (HbA1c) levels (118).

#### Insulin resistance

Much of the literature suggests that HUA induces IR and impaired glucose uptake in various tissues and cells, such as adipose tissue, liver, vascular endothelial cells, and macrophages, through oxidative stress and inflammation (119) (**Figure 3**). The covalent binding of thioredoxin to its interacting protein (TXNIP) in macrophages may suppress ROS clearance by inhibiting the Nrf2/HO-1 pathway and exacerbate IR by activating the IRS2/AKT pathway (120). Obesity has emerged as a notable ground for IR and contributes to the polarization of M1-like adipose tissue macrophages in low-level chronic pathogenic inflammation and IR conditions. SGLT inhibitors have long been used to lower blood glucose levels and treat obese patients. SGLT1 reabsorbs approximately 10% of glucose from urine, while SGLT2 accounts for 90% of the uric glucose reabsorption. Existing data pointed out that SGLT2 inhibition may do good in lowering serum UA and insulin state, as well as reducing the risk of inflammation (121). Sadly, experimental evidence is still lacking on whether all the SGLT2 inhibitors could link UA metabolism and diabetes. However, Tanaka, Y. believed that directly targeting urate transporters may serve as a promising strategy in obesity-associated IR. His team selected dotinurad, a novel URAT1-selective inhibitor, and found out that its mechanism lay in the reduced intracellular UA and ROS levels, decreased Ccl2 and TNF-α in macrophages, and promotion of the browning of white adipocytes and re-browning of brown adipocytes (122). Glut4 is responsible for insulin-mediated glucose uptake in adipocytes and in liver macrophages; UA hinders its translocation into the nucleus, resulting in undermined glucose metabolism via IRS2/PI3K/AKT signaling and AMPK/mTOR signaling during macrophage inflammation (123). DPP-4 effectively upregulates glucose levels and enlarges inflammatory sites, along with affecting normal macrophage function. It stimulates the activity of xanthine dehydrogenase and further the production of UA (124). Nistala et al. uncovered that a Western diet-induced murine model exhibited elevated levels of plasma UA and DPP-4. In addition, the proportion of CD68/ED1^+^ M1 macrophages was enriched, and IL-10 levels were relatively decreased in M2-like macrophages, thereby aggravating diabetic nephropathy (125).

### Others

In addition to the well-known diseases affected by HUA as mentioned above, several studies have attempted to identify plural relatives of HUA. Currently, the U-shaped relationship between UA levels and cancer is widely recognized by educational circles, which means that too much or too little UA can increase the risk of cancer. In a recent cohort investigation involving 444,462 individuals with colorectal cancer, researchers revealed the fact that HUA is a booster of this cancer, especially in women, possibly because of inflammation (126) (**Figure 3**). On the other hand, since UA is also an antioxidant, some insist that HUA can actually relieve cancer. One report on oral cancer demonstrated a descending line of their correlation. They demonstrated that high UA levels, which they considered > 6 mg/dL, halved the probability of illness (127). This is quite shocking, but the limitation may lie in the small number of participants, so most studies still look to the convention. Chronic obstructive pulmonary disease has been linked to HUA in the past few years (**Figure 3**). Nicotine inhalation is one element contributing to the development of this lung problem. One medical group exposed cigarette extract to lung epithelial cells and found that HUA may intensify mitochondrial ROS and apoptosis through the downregulation of peroxiredoxin-2 (128).

## Therapeutic strategies against HUA and associated comorbidities

Increasing evidence supports that UA-lowering therapies are closely associated with inflammation relief, further confirming that HUA is closely linked to the immune system. For routine treatment, we would first encourage improvements in lifestyle across all aspects, such as diet modification, alcohol refinement, moderate workout, and the intake of probiotics and functional supplements, as these can effectively lower UA levels and benefit human health. Nevertheless, UA-targeted medication is necessary if the UA level is uncontrolled and patients do not adhere to previous medical advice. Both strategies are summarized in **Table 1**. High levels of purines in the body caused, caused by an unhealthy diet, can lead to the development of a number of metabolic disorders, such as HUA. Nowadays, three recommended dietary treatments are used to manage HUA and gout: the Dietary Approaches to Stop Hypertension (DASH) diet, the Mediterranean diet, and the low-purine diet. The DASH diet, originally designed to treat hypertension, is rich in vegetables, fruits, and low-fat dairy products, while being reduced in saturated fat and cholesterol (129). Over time, its ability to reduce serum UA levels has been clarified, making it well-suited for both HUA and CVD (130). The Mediterranean diet, which emphasizes high plant-based food consumption, has become popular due to its positive effects on cognitive health, weight loss, and maintaining a healthy body shape. These benefits make it useful for managing metabolic syndromes such as obesity and diabetes. Additionally, its ability to maintain UA levels for 24 months has made it particularly favorable compared to other diets (131). Since purine-rich foods, including red meat, seafood, legumes, and fungi, are a major cause of HUA, it is advisable for HUA patients to control their intake of these foods. Consuming an additional 10 g of refined grains would reduce the risk of HUA by 0.90%. Furthermore, when combined with a time-restricted eating schedule, the role of diet in lowering UA levels will be magnified (132). Probiotics are gaining significant attention for their ability to treat disorders by regulating gut microbiomes, which have recently been shown to maintain host homeostasis and alleviate inflammation, thereby relieving HUA. It is also believed that specific strains of probiotics are responsible for purine degradation (133). Another functional food that deserves attention is inulin, a nondigestible prebiotic fiber found in many edible plants, which affects metabolism. A report on purine-rich diet-induced HUA in quails showed that chicory inulin decreased serum UA levels by decreasing XO levels (134). Lastly, there are several other daily supplements that benefit UA metabolism, such as various tea extracts, vitamin C, vitamin D, and allicin.

**Table 1:** Potential therapies for HUA and associated comorbidities.

Treatment	Study object	Dosage	Effect	Mechanism
Allopurinol (149)	Hypoxanthine and potassium oxonate (PO)-induced HUA male mice	–	↓UA, ADA, creatinine level	XO↓
Allopurinol (150)	High fructose diet-induced Otsuka Long-Evans Tokushima Fatty male rats	10 mg/kg/day	↓UA, hepatic lipid metabolism, curbing inflammation ER stress	XO↓, plasminogen activator inhibitor 1 (PAI-1)↓, TNF-α↓, IRE1-XBP-1↓
Febuxostat (151)	Oxonic acid-induced CKD male rats	3 mg/kg/day	↓UA, urinary albumin levels, oxidative stress	XO↓, Nrf2/HO-1↓
Febuxostat (152)	Male diabetic KK-Ay mice	1 mg/kg/day	↓UA, glucose, glomerular sclerosis	XO↓, IL-1β↓, IL-6↓
Benzbromarone (153)	SU5416 (a VEGF receptor blocker)/hypoxia/normoxia/oxonic acid-induced pulmonary hypertension and HUA male rats	10 mg/kg/day	↓Lung UA levels, ↓right ventricular systolic pressure/occlusive lesion development	Uricosuric agents (URAT1↓)
Benzbromarone (154)	Streptozotocin-induced diabetic male rats	10 mg/kg every 2 days	↓Oxidative stress/retinal inflammation, ↓NLRP3	Uricosuric agents (URAT1↓), IL↓, TNF-α↓, TGF-β↓
Probenecid (155)	*Oat1* knockout mice, *Oat3* knockout mice, *Oat1* and *Oat3* knockout mice	–	↓Urate in plasma, ↑urate in urine	Uricosuric agents (URAT1↓, OAT1↓, OAT3↓)
Losartan (156)	Human renal proximal tubular cells (HK-2 cells)	100 µM	↓AngII and UA-mediated inflammation and oxidative stress	Uricosuric agents (URAT1↓), TLR4↓, MCP1↓, Nox4↓
Losartan (157)	Primary vascular smooth muscle cells from the thoracic aorta of male rats	10^−5^ M	↓MAPK activation triggered by UA and p44/42 MAPK initiation caused by AngII	Uricosuric agents (URAT1↓), AngII type I receptor (AT1R↓)
Tranilast (158)	Mature female *Xenopus laevis* frogs	–	↓[^14^C] urate transport mediated over reabsorptive urate transporters and ↓UA through [^14^C] nicotinate transport	URAT1↓, GLUT9↓, OAT4↓, OAT10↓, and NPT1↓
Topiroxostat (159)	ApoE knockout uninephrectomized CKD mice, low-density lipoprotein-treated HK-2 cells	1 mg/kg/day (animal), 5 µM (cell)	↓Hypercholesterolemia-associated renal dysfunction, NADPH-dependent ROS generation, lipid dysfunction, and inflammation	NF-κB↓, XO↓
Simiao San (160)	Yeast polysaccharide (YP) and PO-induced HUA male mice, THP-1 cells	1, 10 mg/kg/day	↓UA, ↓MSU crystal-induced inflammation, ↑M2 macrophage polarization	NF-κB↓, PI3K/Akt↓
Berberine (161)	RAW 264.7 macrophages, MSU-induced rats	50 mg/kg b·wt (animal)	Exerting an anti-inflammatory and antioxidant effect on MSU crystal-induced inflammation	IL–1β↓, TNF-α↓, Nrf2↓
Leonurine (162)	RAW 264.7 macrophages, MSU-induced male rats (200–250 g)	10 and 20 μM (cell), 15 and 30 mg/kg/day (animal)	↓Cyclooxygenase-2, microsomal prostaglandin E synthase-1 and 5-lipoxygenase, ↓M1 macrophage inflow, ↓MSU-induced inflammation	STAT1↓, NF-κB↓, IL-1β↓, TNF-α↓
Rhein (163)	Gouty arthritis patients, urate crystal-activated THP-1 cells	2.5 μg/mL and 1, 5, and 10 μg/mL	↓The inflammatory response through modulating NLRP3 inflammasome and caspase-1 activity	IL-1β↓, TNF-α↓
*SMILACIS GLABRAE* RHIZOMA water extracts (164)	PO- and MSU-induced chronic HUA and gouty mice	10, 30, and 90 mg/kg/day	↓UA and BUN, mediating UA metabolism, ↓inflammation	XO↓, TNF-α↓, IL-1β↓
Tea water extracts (165)	PO and adenosine-induced HUA male mice (35–40 g, 6 weeks old)	800 mg/kg/day	Regulating UA metabolism, ↓oxidative stress, modulating the gut microbiota (decreased *Firmicutes*/*Bacteroidota* ratio, increased *Ruminococcus* and *Lactobacillus*)	XO↓, URAT1↓, ABCG2↑, OAT1↑, OAT3↑, Nrf2/HO-1↓
Macroporous resin extract of the *Dendrobium candidum* leaf (166)	Modified high purine diet (added with PO and adenine)-induced HUA male rats	4.375 and 17.5 mg/kg/day	Protecting kidney and liver function, modulating UA metabolic reaction, preventing renal inflammation	XO↓, ADA↓, TLR4/NF-κB↓
Lemon water-soluble extract (potassium citrate) (167)	PO-induced HUA male mice (35 g ± 5 g, 8 weeks old)	100, 200, and 400 mg/kg/day	↓UA, urea nitrogen, and creatinine relieve renal function	GLUT9↑, URAT1↓
Spexin (168)	High-fructose diet-induced rats	50 μg/kg/day	↓Metabolic reactions of HUA, hyperglycemia, hyperlipidemia, and ↓inflammation by PPAR-γ and AMPK	IL-6↓, TNF-α↓
Folic acid, zinc (169)	High-purine diet-induced HUA male rats	84 µg/kg/day folic acid, 4 mg/kg/day zinc	↓The formation of UA,↑UA excretion via changing the gut microbiota composition	XO↓, ADA↓
*Lacticaseibacillus rhamnosus* Fmb14 (170)	Chronic purine-induced HUA male mice	10^8^ CFU/day	Modulating the production and excretion of UA, ↑the diversity of gut microbiota, ↑SCFAs in the colon, ↓the inflammation	XO↓, ABCG2↑, URAT1↓, IL-1β↓, IL-18↓, TNF-α↓
Ellagic acid (171)	60% fructose-enriched diet-induced HUA and associated NAFLD male rats	15 mg/kg/day	↓UA levels, protecting liver function, mediating lipid and glucose metabolism by AMPK	XO↓
*Lactiplantibacillus plantarum* (172)	High-nucleoside diet-induced male mice, Caco–2 cell	10^7^ CFU twice/day	↓Urate level by suppressing urate synthesis and excretion	XO↓, purine nucleoside phosphorylase (PNP)↓, ABCG2↓
Inulin (173)	Uox-knockout HUA mice	9.5 g/kg/day	↓UA levels, improving gut leaky and SCFAs production, ↓inflammation	XO↓, ABCG2↑
Vitamin C (174)	PO and adenine-induced hyperuricemic nephropathy rats	10 mg/kg/day	↓Hyperuricemic nephropathy, ↓oxidative stress, ↓renal damage, and inflammation	XO↓, IL-6/JAK2/STAT3↓
Tart cherry powder (175)	PO and adenine-induced HUA male rats	0.17 and 0.50 g/kg·bw	Slightly ↓UA and renal injury	ADA↓
Strictinin (176)	PO-induced HUA male mice	400, 700, and 1,000 mg/kg·bw	↓UA production, ↓hepatic inflammation	XO↓, NF-κB/NLRP3↓
Eggshell membrane (177)	PO-induced HUA male mice	100 and 200 mg/kg/day	↑Renal UA excretion	URAT1↓, OAT1↑, ABCG2↑
Anakinra (140)	MSU crystal-induced HUA mice	200 μg injection	↓The inflammation induced by MSU crystals	IL-1↓

↓, down-regulate; ↑, up-regulate.

If nondrug therapy has little effect on patients, prescriptions are essential to rebalance UA metabolism and uricase activity. Urate-lowering therapy (ULT), the globally approved treatment for HUA, which includes allopurinol and febuxostat, is preferred. Both allopurinol and febuxostat have been shown to have anti-inflammatory effects by adjusting UA metabolism (135, 136). Allopurinol was the first drug approved for treating HUA, but its potentially life-threatening hypersensitivity symptoms, such as Stevens–Johnson syndrome, which primarily occur in the first few months of therapy, are quite dangerous, so it should be used with caution (137). Febuxostat is the drug of choice for treating HUA and CKD, but its adverse effects on the cardiovascular system limit its use (109). Therefore, researchers have begun conducting *in vitro* and *in vivo* studies to address this problem. Fortunately, over the past few decades, they have identified several compounds that target urate transporters involved in the UA metabolism or uricase. In addition to these drugs, herbal medicine, which has long been used to treat HUA and gout in Asia, is gaining recognition as an alternative to well-known Western medications due to its abundant bioactive components and relatively low toxicity, which may pose less harm to the liver and kidneys and cause fewer serious adverse effects. According to TCM theory, HUA is attributed to disorders of spleen and kidney function. It is also believed that patients suffering from HUA tend to be overweight and enjoy a greasy diet, which contributes to the dysfunction of viscera, blood stasis with water retention, and dampness–heat descending. Multiple Chinese herbs and formulas aimed at damping heat and eliminating dampness have been proven to be effective and safe for the treatment of HUA and gout (138). For example, Euodiae fructus is effective in alleviating asymptomatic HUA and gout by restoring UA metabolism and interrupting NLRP3 inflammasome activation, thereby inhibiting the production of proinflammatory cytokines in macrophages (139). Lastly, immunotherapeutic targeting of IL-1, including anakinra and canakinumab, which can directly and precisely block the progression of inflammation, is attracting increasing interest for the treatment of HUA and gout (140, 141). Notably, Chen et al. found that an M2 macrophage-erythrocyte hybrid membrane-disguised bionic nanosized liposome can reprogram the overall inflammatory microenvironment in gouty murine, achieving enzyme-thermo-immunotherapy, which may serve as a biological innovation for future studies (142).

## Analysis of clinical trials of ULT therapies for HUA and related comorbidities

The results of recent clinical trials on UA metabolism disorders, according to the therapies described in **Table 1**, are summarized in **Table 2**. Generally, clinical trials have confirmed the experimental results from preclinical research, with additional complementary points worthy of note. First, although these drugs are tested for achieving inflammatory outcomes, this is not always the case in medical practice. Taking gout as an example, as it is considered a progressive disease, it is wise to use certain medications at its different stages. Starting with an acute gout flare, anti-inflammatory agents, including nonsteroid anti-inflammatory drugs (NSAIDs), colchicine, corticosteroids, and anti-IL-1β medicaments, should be used in this phase. Established gout requires ULT, as these anti-inflammatory remedies can only prevent symptoms, while ULT may clear the production of hidden urate crystals and inhibit the development of chronic gout (143). However, we found that a single administration of ULTs, such as allopurinol and febuxostat, has little effect on inflammatory markers, such as high-sensitivity C-reactive protein (hsCRP), the official clinical index of inflammation. One reason for this observation may be that the anti-inflammation of ULT may not be strong enough to combat the intense inflammatory environment. Another factor is that there are still conflicts regarding whether ULT should be involved in the treatment of acute gout flares. Additionally, many guidelines for gout treatment suggest that once ULT is initiated, it should be administered in a timely and long-term manner until it loses its effectiveness, which makes adherence and persistence quite difficult for patients. However, if nurse-led care is involved in the usage of ULT, the effect may be greatly improved. Thus, from our point of view, in clinical practice, ULTs are indispensable in the treatment of gout. Currently, they may serve as a companion to antiphlogistic solutions, particularly if focused on anti-inflammation, and the combination of two or more ULTs may enhance curative effects. Second, diet, alcohol consumption, and training are tripartite confrontations in improving lifestyles against HUA and related metabolic syndromes. The 2020 ACR guidelines for the management of gout proposed limiting alcohol intake for all gout patients, as alcohol overuse is linked with elevated UA levels (144). Regular, moderate-level exercise is considered an acceptable alternative to lower the risk of HUA. One clinical trial involving 467,976 adults showed that those who exercised an average of 150 min per week, or 30 min a day for more than 5 days a week, at moderate intensity, had an 11% decrease in mortality from HUA. In contrast, HUA shortened the lifespan of inactive men and woman by 4.3 and 5.7 years, respectively (145).

**Table 2:** Clinical trials of urate-lowering therapy for the recovery of metabolic disorders.

Study	At risk	UA-lowering therapy	Finding kidney function and other related effects	Measurement of inflammatory indicators	Limitation
Satpanich et al. (178)	Gout (*n* = 117)	28 days of early or late intervention of allopurinol	↓UA and acute gout flare	The numbers of C-reactive protein (CRP) and white blood cells between the 2 groups show no significant difference.	No significant differences between early and late allopurinol treatment, and mild adverse symptoms
Saag et al. (179)	Gout and a record of CVD or cerebrovascular disease (*n* = 6190)	On average 32 months (85 months at most) of febuxostat (*n* = 3,098) or allopurinol (*n* = 3,092)	↓Tophi, serum UA levels. Febuxostat downregulated UA levels more robustly.	–	No clear association between serum urate levels and death from CV causes in the febuxostat group.
Konishi et al. (180)	HUA elderly with or without a history of CVD (*n* = 1,070)	36 months of febuxostat	↓Serum UA levels, renal damage, CVD risk, mortality rate, and the stake of developing microalbuminuria/mild proteinuria, especially for patients with CVD.	–	Febuxostat does not estimate glomerular filtration rate (eGFR) decrease. For asymptomatic HUA, febuxostat is not superior to the nonfebuxostat group considering the all-cause death.
Hussain et al. (121)	T2DM with adequate glycemic control (defined as HbA1c < 7%), HUA, obesity (*n* = 70)	4 weeks of SGLT-2 inhibitors (dapagliflozin and empagliflozin) or traditional oral antihyperglycemic drugs	↓Serum UA levels in patients with T2DM	–	–
Yan et al. (181)	Gout with UA underexcretion male (*n* = 196)	12 weeks of low-dose benzbromarone or febuxostat	↓Serum urate levels while benzbromarone was more effective than febuxostat.	–	Urolithiasis was observed in both groups.
Stack et al. (182)	CKD1, CKD2, CKD3, albuminuria, T2DM (*n* = 60)	12 weeks of verinurad + febuxostat	↓Serum urate and albuminuria, ↑renal activity	No significant change in serum hsCRP	No major perturbation on eGFR. Higher incidence of diarrhea with verinurad and febuxostat than placebo group.
Bryant et al. (183)	CKD, proteinuria juveniles with or without high blood pressure (*n* = 248)	36 months of losartan or enalapril	Losartan decreased serum UA levels in the first 30 months but increased serum UA levels in the last 6 months. However, enalapril increased serum UA levels over the whole trial in hypertensive patients. Both groups have a beneficial effect on eGFR.	–	The level of eGFR and serum UA are out of sync.
Kohagura et al. (184)	CKD3, hypertension (*n* = 95)	52 weeks of febuxostat or benzbromarone	↓Serum UA level, CKD progression, and inflammation. Febuxostat is more effective for serious CKD patients.	Benzbromarone can reduce hsCRP levels better than febuxostat	No statistical difference in the change in eGFR, minor and similar adverse events between groups
Kataoka et al. (185)	CKD3, asymptomatic HUA with or without proteinuria (*n* = 395)	108 weeks of febuxostat	↑Kidney function among CKD3 patients with asymptomatic HUA without proteinuria over a long-term	–	Almost no effect on eGFR in the first 24 weeks
Jalal et al. (186)	CKD3 (*n* = 80)	12 weeks of allopurinol	↓UA levels and ↑endothelial function although not statistically identified.	Cannot decline IL-6, MCP-1, however, ↓NF-kB	–
Tanaka et al. (187)	HUA (*n* = 40)	3 weeks of rasburicase	↓UA levels sharply	↑IL-6	Unable to detect apparent effect in the tested inflammatory markers and oxidative stress
Alshahawey et al. (188)	HUA, long-term hemodialysis (*n* = 57)	2 months of febuxostat	↓Serum UA levels and vascular dysfunction through lowering blood pressure	↓hsCRP and white blood cells	–
Takir et al. (189)	Asymptomatic HUA (*n* = 73)	3 months of allopurinol	↓Serum UA levels, ↑glucose metabolism	↓hsCRP	–
Mackenzie et al. (190)	Gout with a history of cardiovascular disease (*n* = 6,128)	8 years of febuxostat or allopurinol	Febuxostat is a safer choice for treating this kind of disease than allopurinol.	–	Using enduring allopurinol or febuxostat may increase the incidence rate of tumors.
Li et al. (191)	Gout (*n* = 983)	24 weeks of fenofibrate	↓Serum urate and TG levels	–	Nephrotoxicity, if taken 3 weeks or more, is a mild elevation in serum creatinine.
Kahl et al. (192)	T2DM (*n* = 84)	6 months of empagliflozin	↓Serum UA levels, weight loss, and liver fat decline	–	No improvement in insulin sensitivity
Rai et al. (193)	Gout (*n* = 44,444)	26 years of DASH diet	↓Gout risk and as the adherence goes further, the prevalence continuously goes down	–	–
Yokose et al. (131)	BMI > 27 kg/m^2^ or T2DM or coronary heart disease (*n* = 76)	2 years of Mediterranean diet	↓Rapidly serum urate levels and body weight, ↑renal function, and insulin sensitivity within 6 months and a long-standing maintenance effect over the duration of 24 months	–	–
Brymora et al. (194)	CKD2, CKD3 (*n* = 28)	6 weeks of low-fructose diet	↓Serum UA without a significant difference, fasting serum insulin levels, diastolic blood pressure and mean arterial pressure	↓hsCRP and soluble intercellular adhesion molecule1 levels	Slight but not enormous reduction in urinary and fractional UA excretion. No significant difference in eGFR, TNF-α, MCP-1, and TGF-β levels compared with a regular diet
Hou et al. (195)	HUA without gout (*n* = 186)	1 year of low- or moderate-intensity exercise intervention	Both decreased serum UA levels but a greater reduction in serum UA levels in the moderate-intensity exercise group (this is only a hypothesis because this trial is still in progress)	–	–
Belanger et al. (196)	Elevated blood pressure or hypertension (*n* = 163)	6 weeks of 3 different DASH-style diets: a carbohydrate-rich (CARB) diet, a protein-rich (PROT) diet, and an unsaturated fat-rich (UNSAT) diet	All decrease serum urate levels with the PROT diet (especially plant-based protein) being the most effective, and this kind of PROT diet can effectively help individuals maintain relatively good health. For patients with gout, the ability to lower UA levels is more obvious.	–	–
Zhang et al. (197)	CKD1-3a (*n* = 84)	12 weeks of spironolactone and low- or medium-sodium diet	Low- and medium-dose sodium may decrease urine protein and spironolactone may further promote these effects	–	3 patients who ate high-potassium food in the low-sodium + spironolactone group had increased blood potassium levels, no significant change in UA and eGFR levels
Juraschek et al. (198)	Prehypertension or stage 1 hypertension (*n* = 103)	DASH-sodium diet: DASH diet + 30 days of 60, 120, or 180 mmol of sodium	↓UA levels, and this effect is attributed to both the diet pattern and sodium intake but the specific DASH diet dominates it	–	Increasing sodium intake from medium to high concentration has no practical effect on lowering UA levels.
Kawakami et al. (199)	Male with alcohol consumption (*n* = 10)	Catechin-rich green tea	Mediating UA metabolism	–	Failed to detect the peak time of UA, xanthine, and hypoxanthine after alcohol ingestion
Yu et al. (138)	HUA, gout (dampness-heat pouring downward pattern) (*n* = 72)	4 weeks of Yellow-dragon Wonderful-seed Formula or Yellow-dragon Wonderful-seed Formula + gypsum	↓Urine urate and the addition of gypsum amplified this effect	–	No obvious change in serum UA levels
Xi et al. (200)	Impaired spleen function, CKD2–CKD3 (*n* = 100)	6 months after Qi Gui Yi Shen’s decoction	Significantly improving CKD symptoms, ↑creatinine clearance rate	–	–
Lukenda Zanko et al. (201)	NAFLD (*n* = 311)	12 months (360 ± 7 days) of vitamin D3	↓UA, ↑gamma-glutamyl transferase levels	No apparent effect on CRP levels	–
Panahi et al. (92)	NAFLD (*n* = 102)	8 weeks of curcumin	↓UA levels, ↑ lipid metabolism	–	–
Botson et al. (202)	Gout (*n* = 14)	Maximum 52 weeks of pegloticase after 4 weeks of methotrexate	↓UA levels, ↓tophi	–	Gout flares were comparatively frequent during pegloticase intervention.
Miner et al. (203)	Gout (*n* = 8)	Lesinurad tablets (200, 400, or 600 mg)	↓UA levels in a dose-dependent manner, ↓URAT1 and OAT4, ↓OAT1, and OAT3	–	–
Borgi et al. (204)	Nonhypertensive and obese individuals with UA levels ≥ 5 mg/dL (*n* = 149)	8 weeks of probenecid or allopurinol	↓UA levels while allopurinol had a marginal advantage	–	Unable to improve endothelial dysfunction
Waldman et al. (205)	T2DM (*n* = 9,795)	About 5 years of fenofibrate	↓UA levels almost halved the rate of first gout flare	–	–
Wada et al. (206)	Diabetic nephropathy, HUA, micro-albuminuria (*n* = 65)	28 weeks of topiroxostat (40, 80, 120, and 160 mg/day)	↓UA levels, ↑eGFR	–	Mild elevations of AST and ALT levels
Tausche et al. (207)	Gout who cannot take allopurinol or febuxostat (*n* = 214)	6 months of lesinurad	↓UA levels for a longer time	–	Possibility of serious serum creatinine elevations and increased renal failure events
Solomon et al. (141)	Experienced a myocardial infarction before 1 month of enrollment and hsCRP ≥ 19.1 nmol/L (*n* = 10,061)	7 years of canakinumab (50, 150, and 300 mg) every 3 months	↓Cumulative incidence of gout attacks	↓hsCRP level dose-dependently	No significant effect on serum UA levels compared with the placebo group
Wang et al. (208)	Gout (*n* = 282)	12 weeks of tart cherry supplementary citrate	↓Serum urate levels, alkalization of urine, and glucose metabolism dysfunction	↓CRP notably	–
Yu et al. (209)	Refractory gout (*n* = 64)	3 months of febuxostat or febuxostat + diacerein	The combination of 2 drugs improved the urate-lowering ability and gout flare incidence of febuxostat alone.	The addition of diacerein further downregulated IL-1β	–

↓, down-regulate; ↑, up-regulate.

## Conclusion and discussion

Increasing evidence suggests that high levels of UA and its crystalline form are major causes of many metabolic comorbidities, including gout, CKD, IR, CVD, and NAFLD. During the onset of these diseases, UA elicits an inflammatory response by activating the innate immune system. Subsequently, macrophages and their precursor cells, monocytes, are activated, influencing the progression of diseases through the involvement of NF-κB, the NLRP3 inflammasome, and TLR4, all of which contribute to the release of IL-1. In summary, IL-1 molecules (IL-1α and IL-1β) are prototypic proinflammatory cytokines that play a role in inflammatory processes by combining to the IL-1 receptor (IL-1R), whereas their antagonist, IL-1Ra, binds to IL-1R1, inhibiting the integration of IL-1R1 with IL-1α and IL-1β (146). Additionally, among these diseases, UA is sometimes the direct pathogenic factor and, at other times, aggravates the illness indirectly. For example, during the progression of HUA-related CVD, the first target cells are endothelial cells; otherwise, UA acts as a trigger, worsening the onset of disease via activation of the innate immune system.

Moreover, due to changes in many aspects, an increasing number of people are suffering from HUA, which may propel greater demand for further research and the repositioning of ULT (147). Many drug and nondrug therapies are listed in the table, and recent clinical trials have provided a complete overview of these treatments. However, real-life drug administration for HUA remains a significant challenge, and unfortunately, we still rely on ULT rather than newly created drugs for various reasons, including complex pathogenesis of HUA, toxicity of drugs, contraindications between drugs, differences in efficacy, and differences in individual physiques, despite the fact that ULT may also cause certain adverse effects. Therefore, based on the current situation, ULT remains the pole of HUA treatment, and further research is needed to develop safe and effective drugs or identify alternative therapies capable of achieving similar curative effects to alleviate HUA and maintain UA homeostasis.

In summary, this review briefly discusses how UA or urate crystals contribute to diseases, as well as the changes in innate immune cells, particularly macrophages, under asymptomatic HUA or HUA-induced conditions, based on previous studies. It aims to provide theoretical support for research. It should be solemnly declared that HUA may lay the foundation for other illnesses, such as cancer and chronic obstructive pulmonary disease, which we include in this writing to provide a more holistic picture. Of note, there are some humble suggestions we wish to share. First, although several studies have reported the relation between HUA and its related metabolic diseases and macrophage polarization, there are still likely mechanisms and related fields that have not been clearly elucidated. Therefore, future large-scale studies are expected. Second, while many clinical trials on drugs to alleviate HUA exist, trials related to improved lifestyle, diet regulation, and functional food intake are lacking. Third, there is an interesting issue regarding the mechanism of asymptomatic HUA, as most research has focused on HUA-related diseases but seldom on asymptomatic HUA itself. Hopefully, this imbalance will be addressed in the years to come. Finally, metabolic abnormalities in one disease can promote the development of other diseases. For example, one Korean study reported that NAFLD is at a higher risk for developing HUA and CVD, while another study demonstrated similar molecular pathways shared by both glucose mal-metabolism and lipoprotein oxidation under HUA conditions (104, 148). These types of trials are gaining more attention and appreciation. Thus, perhaps in our laboratory, it is reasonable to determine the proper model to study these complex disease patterns.
