# Supplementary figures and images for: Hyperuricemia-induced complications: dysfunctional macrophages serve as a potential bridge
Source: Front Immunol. 2025 Jan 28;16:1512093. doi: 10.3389/fimmu.2025.1512093 (PMC11810932; doi:10.3389/fimmu.2025.1512093)

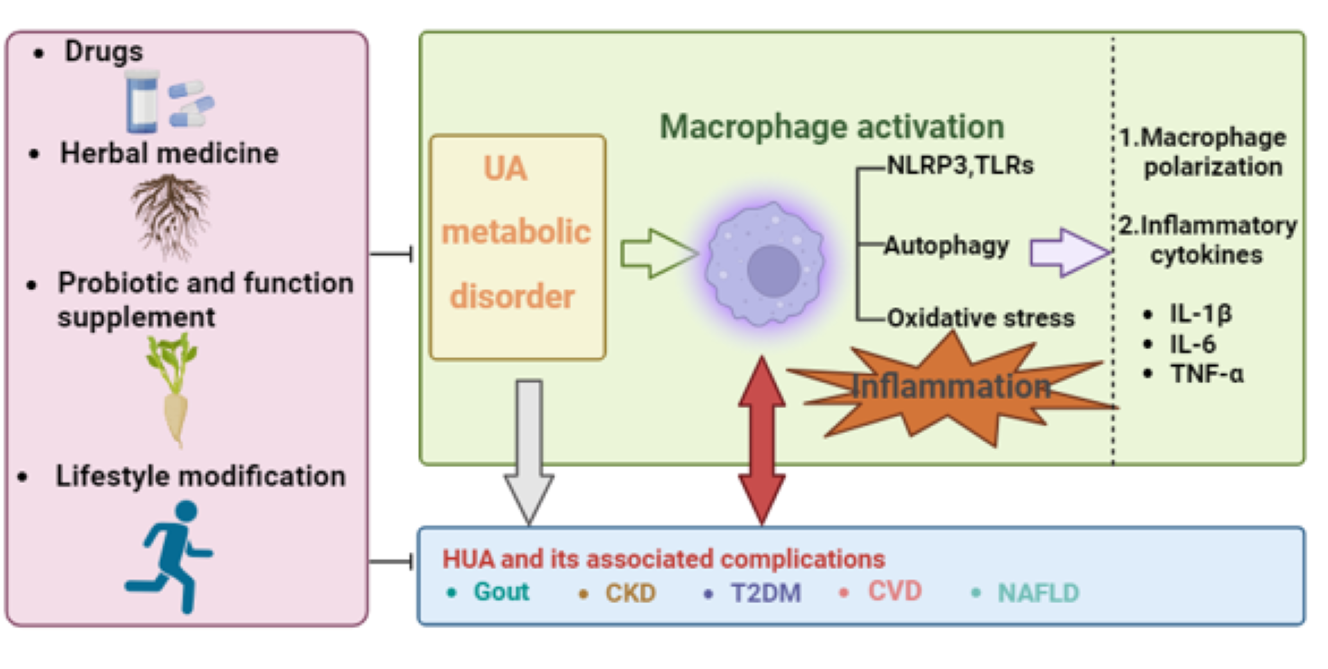

Supplement: Supplementary file 1 [file Image1.tif]
