# Supplementary material for: Hyperuricemia-induced complications: dysfunctional macrophages serve as a potential bridge
Source: Front Immunol. 2025 Jan 28;16:1512093. doi: 10.3389/fimmu.2025.1512093 (PMC11810932; doi:10.3389/fimmu.2025.1512093)
Supplement: Supplementary file 2 [file Table1.docx]

Supplementary Table1. Research on the macrophage activity induced by sUA and MSU crystal

|  | soluble UA | MSU crystal |
| --- | --- | --- |
| Main disease situation | asymtomatic HUA | gout |
| Effect on macrophage activity | aggravate inflammation; alleviate inflammation(anti-oxidation) | aggravate inflammation |
| Common mechanism | TLRs signaling and NLRP3 inflammasome | |
| Representative examples | Epigenic modification→sUA→Akt/mTOR/PRAS40→NF-κB→ NLRP3 inflammasome→IL-1β↑[1] | MSU crystal→(post-translational modification, co-factors of NLRP3 inflammasome, ROS, K+ efflux, glycosis, DRG neurons)→TLR→NF-κB→NLRP3 inflammasome→IL-1β, pyroptosis↑├Autophagy[2-6] |
|  | Naip1→sUA→TLR→NF-κB→NLRP3 inflammasome→IL-1β↑[7] | MSU crystal→ (TREM-1 interact with) TLR4/MyD88/NF-κB→IL-1β, TNF-α, IL-8↑[8, 9] |
|  | sUA→TLR→NF-κB→TNF-α, CD11c↑[10] | MSU crystal→RIPK3/MLKL-dependent necroptosis of macrophage[11] |
|  | sUA→ PI3K/AKT →ROS→NLRP3 inflammasome→IL-1β↑[12] | MSU crystal→JNK→Jun→M1 macrophage polarizaton↑[13] |
|  | sUA→ROS, IL-6, pro-inflammatory polarization of macrophages↓[14] | MSU crystal→CTSD-associated autophagy-lysosome pathway↓→apoptosis↑[15] |

Reference

1. Crişan, T.O., et al., *Uric acid priming in human monocytes is driven by the AKT-PRAS40 autophagy pathway.* Proc Natl Acad Sci U S A, 2017. **114**(21): p. 5485-5490.

2. Biasizzo, M. and N. Kopitar-Jerala, *Interplay Between NLRP3 Inflammasome and Autophagy.* Front Immunol, 2020. **11**: p. 591803.

3. Ha, J., et al., *SERTAD1 initiates NLRP3-mediated inflammasome activation through restricting NLRP3 polyubiquitination.* Cell Rep, 2024. **43**(2): p. 113752.

4. Agrawal, M., et al., *TET2-mutant clonal hematopoiesis and risk of gout.* Blood, 2022. **140**(10): p. 1094-1103.

5. Li, P., et al., *Kv1.5 channel mediates monosodium urate-induced activation of NLRP3 inflammasome in macrophages and arrhythmogenic effects of urate on cardiomyocytes.* Mol Biol Rep, 2022. **49**(7): p. 5939-5952.

6. Renaudin, F., et al., *Gout and pseudo-gout-related crystals promote GLUT1-mediated glycolysis that governs NLRP3 and interleukin-1β activation on macrophages.* Ann Rheum Dis, 2020. **79**(11): p. 1506-1514.

7. Braga, T.T., et al., *Sensing soluble uric acid by Naip1-Nlrp3 platform.* Cell Death Dis, 2021. **12**(2): p. 158.

8. He, Y., et al., *Inhibition of Triggering Receptor Expressed on Myeloid Cell-1 Alleviates Acute Gouty Inflammation.* Mediators Inflamm, 2019. **2019**: p. 5647074.

9. Ahn, H., et al., *Nonsaponin fraction of Korean Red Ginseng attenuates cytokine production via inhibition of TLR4 expression.* J Ginseng Res, 2019. **43**(2): p. 291-299.

10. Crișan, T.O., et al., *Soluble uric acid primes TLR-induced proinflammatory cytokine production by human primary cells via inhibition of IL-1Ra.* Ann Rheum Dis, 2016. **75**(4): p. 755-62.

11. Zhong, C.S., et al., *Gout-associated monosodium urate crystal-induced necrosis is independent of NLRP3 activity but can be suppressed by combined inhibitors for multiple signaling pathways.* Acta Pharmacol Sin, 2022. **43**(5): p. 1324-1336.

12. Ives, A., et al., *Xanthine oxidoreductase regulates macrophage IL1β secretion upon NLRP3 inflammasome activation.* Nat Commun, 2015. **6**: p. 6555.

13. Cobo, I., et al., *Monosodium urate crystals regulate a unique JNK-dependent macrophage metabolic and inflammatory response.* Cell Rep, 2022. **38**(10): p. 110489.

14. Gnemmi, V., et al., *Asymptomatic Hyperuricemia Promotes Recovery from Ischemic Organ Injury by Modulating the Phenotype of Macrophages.* Cells, 2022. **11**(4).

15. Chen, Y.H., et al., *Gouty arthritis involves impairment of autophagic degradation via cathepsin D inactivation-mediated lysosomal dysfunction that promotes apoptosis in macrophages.* Biochim Biophys Acta Mol Basis Dis, 2023. **1869**(6): p. 166703.
